# Supplementary material for: From sequence to enzyme mechanism using multi-label machine learning
Source: BMC Bioinformatics. 2014 May 19;15:150. doi: 10.1186/1471-2105-15-150 (PMC4229970; doi:10.1186/1471-2105-15-150)
Supplement: Additional file 2 — Java code of ml2db. Additional file ml2db_code.tar.gz contains the Java source code to run the multi-label machine learning experiments and save the results to database. The code’s Javadoc is included. [file 1471-2105-15-150-S2.zip › additional file 2/ml2db/utils/doc/uk/ac/standrews/utils/main/database/class-use/DbConn.html]

Uses of Class uk.ac.standrews.utils.main.database.DbConn


JavaScript is disabled on your browser.


- Overview
- Package
- Class
- Use
- Tree
- Deprecated
- Index
- Help

- Prev
- Next

- Frames
- No Frames

- All Classes

## Uses of Class uk.ac.standrews.utils.main.database.DbConn

- Packages that use DbConn

  | Package | Description |
  | --- | --- |
  |  |  |
  | --- | --- |
  | uk.ac.standrews.utils.main.database |  |
  | uk.ac.standrews.utils.test.database |  |
- - ### Uses of DbConn in uk.ac.standrews.utils.main.database

    Methods in uk.ac.standrews.utils.main.database that return DbConn

    | Modifier and Type | Method and Description |
    | --- | --- |
    |  |  |
    | --- | --- |
    | `DbConn` | DbManager.`getDbConnection()` |
    | `DbConn` | DbManaged.`getDbConnection()` |

    Methods in uk.ac.standrews.utils.main.database with parameters of type DbConn

    | Modifier and Type | Method and Description |
    | --- | --- |
    |  |  |
    | --- | --- |
    | `static boolean` | DbUtils.`elementExistsInTable(java.lang.String table, java.lang.String column, java.lang.String element, DbConn dbConn)` Get if an element exists in a table column |
    | `static boolean` | DbUtils.`elementsExistsInTable(java.lang.String table, java.lang.String column1, java.lang.String element1, java.lang.String column2, java.lang.String element2, DbConn dbConn)` Get if two elements exists in the same table row |
    | `static boolean` | DbUtils.`existsInTable(java.lang.String query, DbConn dbConn)` |
    | `static java.sql.ResultSet` | DbUtils.`getResultSet(java.lang.String sql, DbConn dbConn)` Executes an sql query returning a result set |
    | `static boolean` | DbUtils.`insert(DbConn conn, java.lang.String insertSql)` Executes sql insert and returns whether the insert was successful. |
    | `static boolean` | DbUtils.`similarElementExistsInTable(java.lang.String table, java.lang.String column, java.lang.String element, DbConn dbConn)` |
  - ### Uses of DbConn in uk.ac.standrews.utils.test.database

    Methods in uk.ac.standrews.utils.test.database that return DbConn

    | Modifier and Type | Method and Description |
    | --- | --- |
    |  |  |
    | --- | --- |
    | `static DbConn` | DbConnPropsTest.`getDbConnProps()` |

- Overview
- Package
- Class
- Use
- Tree
- Deprecated
- Index
- Help

- Prev
- Next

- Frames
- No Frames

- All Classes
